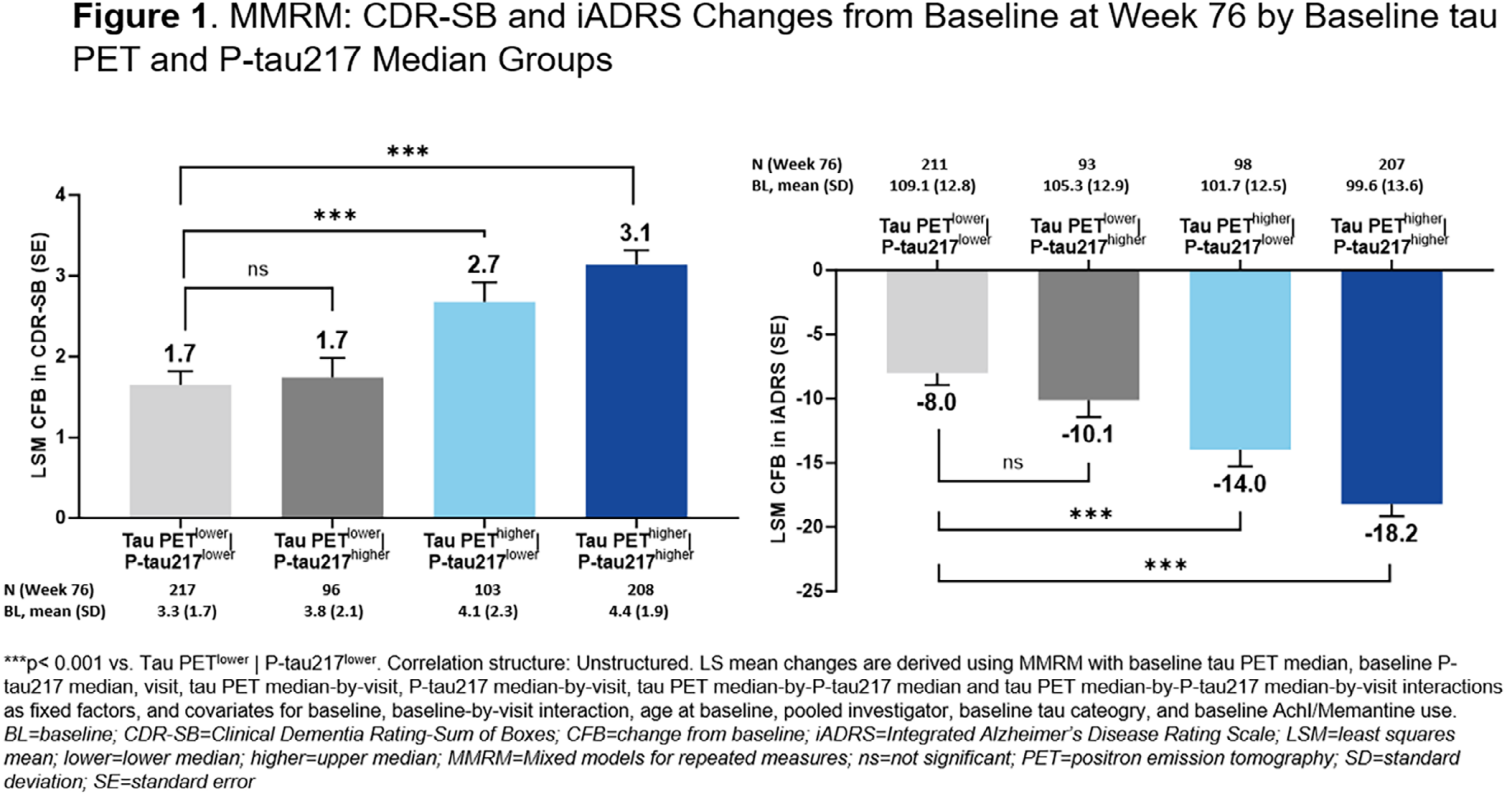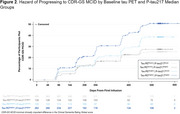# Tau PET and Plasma *p*‐tau217 as Independent and Synergistic Predictors of Clinical Progression in Early Symptomatic AD Patients With Amyloid and Tau Pathology

**DOI:** 10.1002/alz70856_106363

**Published:** 2026-01-10

**Authors:** Min Jung Kim, Amanda Morris, Leonardo Iaccarino, Michael Pontecorvo, Sergey Shcherbinin, John R. Sims, Dawn A. Brooks, Emily C. Collins, Mark A. Mintun, Ming Lu

**Affiliations:** ^1^ Eli Lilly and Company, Indianapolis, IN, USA

## Abstract

**Background:**

Blood‐based biomarkers have significant potential to aid in the diagnosis of Alzheimer's disease (AD), providing a more accessible option than cerebrospinal fluid testing or positron emission tomography (PET). The objective of this study is to evaluate the prognostic value of a plasma *p*‐tau217 immunoassay and tau PET imaging with respect to clinical progression.

**Method:**

Placebo‐treated, early symptomatic AD patients (all amyloid‐positive by amyloid PET) in the TRAILBLAZER‐ALZ 2 study (NCT04437511) were analyzed. Patients (starting cohort *N* = 857) were split into below and above median groups based on median values of baseline flortaucipir tau PET signal evaluated by global AD‐signature region‐of‐interest standardized uptake value ratios (Tau PET^lower^, Tau PET^higher^) and baseline plasma *p*‐tau217 levels (*p*‐tau217^lower^, *p*‐tau217^higher^). The following clinical measures were assessed at Week 76: least squares mean change from baseline in Clinical Dementia Rating ‐ Sum of Boxes (CDR‐SB) and integrated AD Rating Scale (iADRS). Time‐to‐event for the minimal clinically important difference in CDR Global score (CDR‐GS MCID), defined as any increase in CDR‐GS for 2 consecutive visits, was estimated using Kaplan‐Meier analyses. Hazard ratios (HR), 95% confidence intervals (CI), and *p*‐values were calculated using Cox proportional hazards model. These analyses are detailed in the figures.

**Result:**

At Week 76, significant differences compared to baseline in both CDR‐SB and iADRS were observed between patients with baseline Tau PET^higher^|P‐tau217^lower^ or Tau PET^higher^|P‐tau217^higher^ and those with baseline Tau PET^lower^|P‐tau217^lower^ (*p* <0.001); although not statistically significant, a higher rate of clinical progression was observed in Tau PET^lower^|P‐tau217^higher^ than Tau PET^lower^|P‐tau217^lower^ (Figure 1). An increased risk of progressing to CDR‐GS MCID was observed in patients with baseline Tau PET^higher^|P‐tau217^higher^ (HR [95% CI]=1.9 [1.2‐3.1]; *p* <0.05) compared with those with baseline Tau PET^lower^|P‐tau217^lower^ (Figure 2).

**Conclusion:**

Patients with both higher baseline Tau PET and *p*‐tau217 showed a statistically significant clinical worsening at Week 76 compared with those with both lower baseline Tau PET and *p*‐tau217. Results suggest that, although baseline values of tau PET imaging and *p*‐tau217 alone predicted clinical worsening at Week 76, using both tau PET and *p*‐tau217 provided a better prognostic value than tau PET or *p*‐tau217 alone in patients with early symptomatic AD.